# Comprehensive treatment for *ROS1* ‐overexpressed pulmonary sarcomatoid carcinoma: A case report

**DOI:** 10.1002/ccr3.7365

**Published:** 2023-05-18

**Authors:** Wei Sun, Xinlian Wang, Qifeng Shi, Xiao Li, Chaobo Chen

**Affiliations:** ^1^ Department of Respiratory and Critical Care Xishan People's Hospital of Wuxi City Wuxi China; ^2^ Department of Cardiothoracic Surgery Xishan People's Hospital of Wuxi City Wuxi China; ^3^ Department of Pathology Xishan People's Hospital of Wuxi City Wuxi China; ^4^ Department of Pathology the First Affiliated Hospital of Nanjing Medical University Nanjing China; ^5^ Department of General Surgery Xishan People's Hospital of Wuxi City Wuxi China; ^6^ Department of Hepatic‐Biliary‐Pancreatic Surgery The Affiliated Drum Tower Hospital of Nanjing University Medical school Nanjing China; ^7^ Department of Immunology, Ophthalmology & ORL Complutense University School of Medicine Madrid Spain

**Keywords:** antiangiogenetic therapy, chemotherapy, genetic testing, non‐small cell lung cancer (NSCLC), pulmonary sarcomatoid carcinoma, ROS1

## Abstract

**Key Clinical Message:**

In conclusion author highlights the tumor cell genetic testing or molecular pathological diagnosis plays a key role in the individualized treatment of PSC, which could benefit patients with advanced PSC.

**Abstract:**

An uncommon form of non‐small‐cell lung cancer (NSCLC) with a poor prognosis is pulmonary sarcomatoid carcinoma (PSC). Surgical resection is currently the preferred treatment, but guidelines for adjuvant chemotherapy have not yet been established, especially for the advanced stage. The development of molecular subgroups in the field of tumors may be advantageous to advanced PSC patients with the ongoing progress of genomics and immunology. A 54‐year‐old man presented to Xishan People's Hospital of Wuxi City with recurrent intermittent dry cough with fever for 1 month. Further examinations suggested the diagnosis of PSC occupying almost the entire right interlobar fissure area combined with malignant pleural effusion (Stage IVa). Pathological examination confirmed the diagnosis of PSC with *ROS1* overexpressing via genetic testing. However, after three cycles of chemo‐, antiangiogenetic‐ and immunochemical therapy, the lesion was localized, and pleural effusion disappeared, the patient subsequently received an operation which was performed as R0 resection. Unfortunately, the patient became deteriorated quickly followed by extensive metastatic nodules in the thoracic cavity. Although the patient continued to receive chemo‐ and immunochemical‐therapy, it did not limit the progress of the tumor, leading to widespread metastasis, and eventually died of multiple organ failure. For PSC patients with Stage IVa, chemo‐, antiangiogenetic‐ and immunochemical‐therapy performs well in clinical efficacy, and comprehensive panel‐based genetic testing may offer PSC patients a somewhat better prognosis. However, blindly implementing surgical treatment may bring harm to the patient and affect long‐term survival. It's essential to know the surgical indications precisely by NSCLC guidelines.

## Graphical abstract

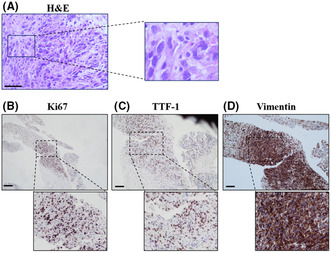

## 1 INTRODUCTION

The non‐small cell lung cancer (NSCLC) known as pulmonary sarcomatoid carcinoma (PSC) is a rare disease that is characterized by the development of sarcomatoid‐like changes and poor differentiation of heterotypic cells. PSC was frequently detected at a later stage, exhibited high levels of aggression, and had a poor prognosis regardless of stage.^1^, ^2^ As classified, PSC was categorized into five subtypes, including pleomorphic carcinoma, spindle cell carcinoma, giant cell carcinoma, carcinosarcoma, and pulmonary blastoma.^1^ Epidemiologically, the incidence of PSC ranges from approximately 0.1% to 0.4% in NSCLC.^3^ Surgical resection is currently the preferred treatment, but guidelines for adjuvant chemotherapy have not yet been established, especially for the advanced stage.^4^ However, the benefit of adjuvant chemotherapy in surgically treated PSC patients in this rare cancer remains controversial. Additionally, immuno‐targeted therapy may be important in the management of PSC. In unresectable diseases, patients can usually only receive first‐line chemotherapy, but often these patients have poor responses.^5^ In fact, in recent years, due to the clinical application of the comprehensive treatment, the mortality rate of NSCLC has been significantly decreased, and the survival rate has been greatly improved, which is closely related to the clinical research progress of immunotherapy and/or targeted therapy. This was due to the clinical‐specific inhibitory effect of EGFR and ALK inhibitors on the corresponding high‐expressing gene tumor patients.^1^, ^6^ Moreover, newly discovered pharmacogenetic drivers, such as *ROS1*, *RET*, *NTRK1‐3*, *BRAF*, *MET*, *TP53*, and *ERBB2*, as well as the implementation of immunotherapy and early detection technologies, may further improve the 5‐year survival rate of NSCLC, not only PSC^1^. In this case, we discuss our experience with the diagnosis and therapy of a PSC case with overexpressed *ROS1* and *PD‐L1* using chemo‐, antiangiogenetic‐, and immunochemical therapy, also link a literature review. Therefore, the new understanding and experience may serve as a foundation for future PSC diagnoses and therapies.

## 2 CASE REPORT

A 54‐year‐old male patient had 30 pack‐years of cigarette smoking and intermittent dry cough that persisted for 1 month, especially when changing body positions, without any significant weight loss, less sputum without blood, and fluctuating body temperature between 37.3 and 38.0°C (axillary temperature). There's no effect after being treated with the Suhuang Zhike capsule (a traditional Chinese medicine used to relieve cough and resolve phlegm). Therefore, he went to Xishan People's Hospital of Wuxi City for further diagnosis and treatment on May 2, 2022. The patient's information was disclosed with consent following a discussion by the hospital ethics committee (No. xs2022ky012).

The physical examination and initial vital signs were as follows: On admission, body temperature, 36.7°C (axillary temperature), pulse rate, 99 beats/min; respiratory rate, 17 breaths/min; and blood pressure, 115/86 mmHg. His height and body weights were 178 cm and 67.5 kg. His familial history was unremarkable. Laboratory examinations showed blood cell analysis, liver function, coagulation function, and serum tumor biomarkers within the normal range (Table 1). Other Laboratory examinations showed a negative result (Table 2).

**TABLE 1:** Characteristic and clinical features of patient.

Items	Results
WBC (^9/L)	8.98
RBC (^9/L)	3.91
Hemoglobin (g/L)	125
Neutrophils (^9/L)	6.5
Platelet (^9/L)	271
CRP (mg/L)	15.9
ESR (mm/h)	75
Procalcitonin (luminescence method, ng/ml)	0.14
DBIL (μmol/L)	3.5
TBIL (μmol/L)	11.7
ALT (u/L)	15
AST (u/L)	12
ALP (u/L)	69
GGT (u/L)	25
ALB (g/L)	34.1
TG (mmol/L)	1.15
CHOL (mmol/L)	3.94
D‐dimer (μg/mL)	0.69
PT (S)	11.7
APTT (S)	32.1
TT (S)	17.9
INR	0.97
AFP (ng/mL)	2
CEA (ng/mL)	1.29
CA199 (U/mL)	<2.00
CA 125 (U/mL)	17.9
CA 153 (U/mL)	10.5
CYFRA211 (ng/mL)	1.79
FPSA (ng/mL)	0.363
PSA (ng/mL)	2.223
S‐SCC (ng/mL)	0.7
S‐NSE (ng/mL)	14.9

Abbreviations: AFP, alpha fetoprotein; ALB, albumin; ALP, alkaline phosphatase; ALT, alanine aminotransferase; APTT, activated partial thromboplastin time; AST, aspartate aminotransferase; CA 125, cancer antigen 125; CA153, Cancer antigen 15–3; CA199, Carbohydrate antigen 19–9; CEA, carcinoembryonic antigen; CHOL, serum total cholesterol; CRP, C reactive protein; CYFRA211, cytokeratin 19 fragment; DBIL, Direct bilirubin; ESR, erythrocyte sedimentation rate; FPSA, free prostate antigen; GGT, Gamma‐glutamyl Transferase; INR, international normalized ratio; PSA, prostate antigen; PT, prothrombin time; RBC, Red blood cell; S‐NSE, Serum neuron specific enolase; S‐SCC, squamous cell carcinoma antigen SCC; TBIL, total bilirubin; TG, triglycerides; TT, thrombin time; WBC, white blood cell.

**TABLE 2:** Laboratory special examination.

Items	Results
Tuberculin test	Negative
T‐SPOT test	Negative
Galactomannan test	Negative
BDG test	Negative
T‐lymphocyte subsets (flow cytometry)	Normal

*Note*: BDG Test, fungal detection tests, galactomannan and 1,3‐β‐d‐glucan test.

**FIGURE 1:**
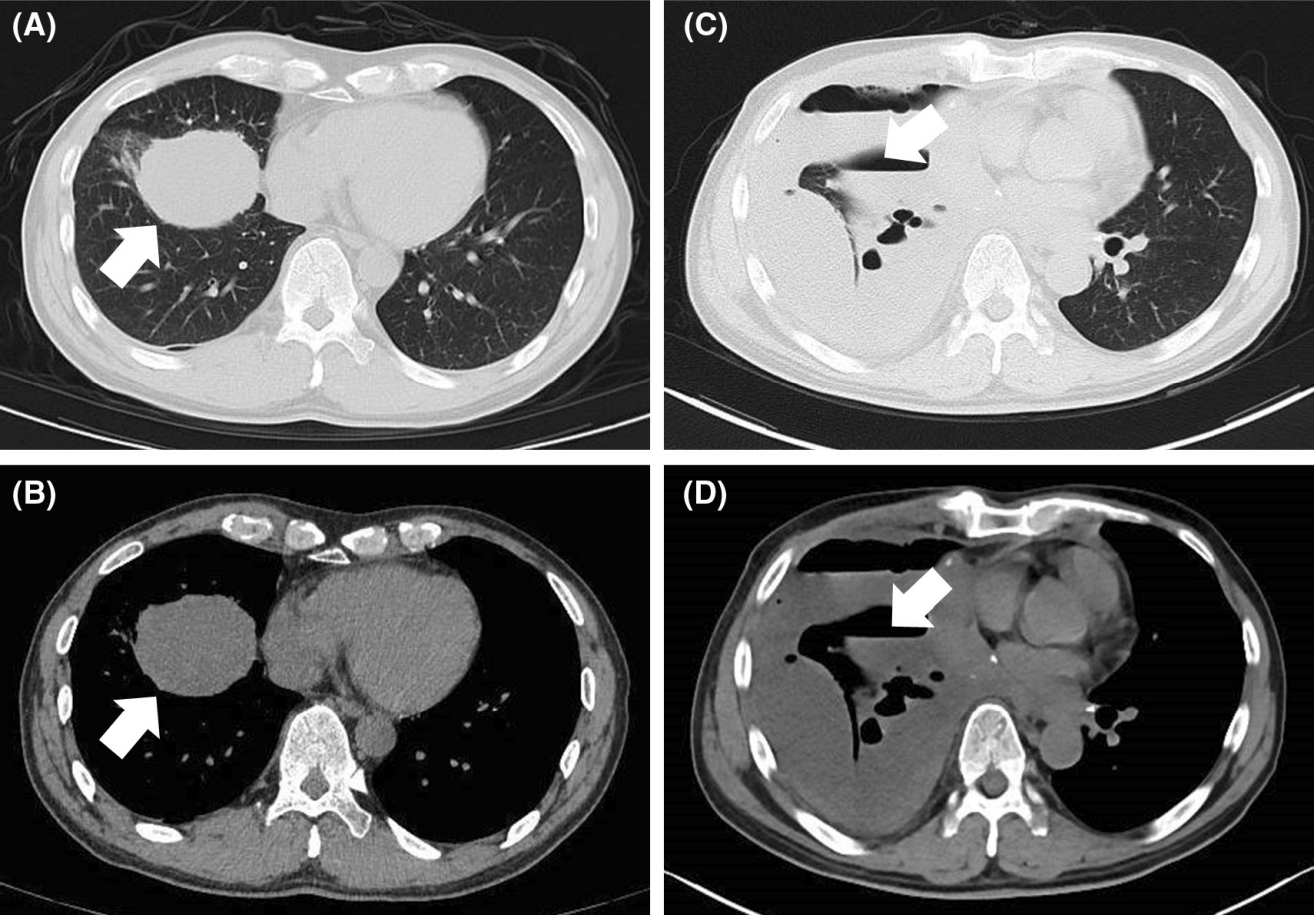
The features of chest CT image corresponding to the patient's condition. (A) A larger pulmonary nodule was obviously detected as arrow shown in the lung window. (B) A larger pulmonary nodule was detected as arrow shown in the mediastinal window. (C,D) The right pleural effusion was accompanied by the formation of air‐fluid level, which was partially encapsulated, and the right lung was infected with atelectasis after the first operation as arrow shown.

**FIGURE 2:**
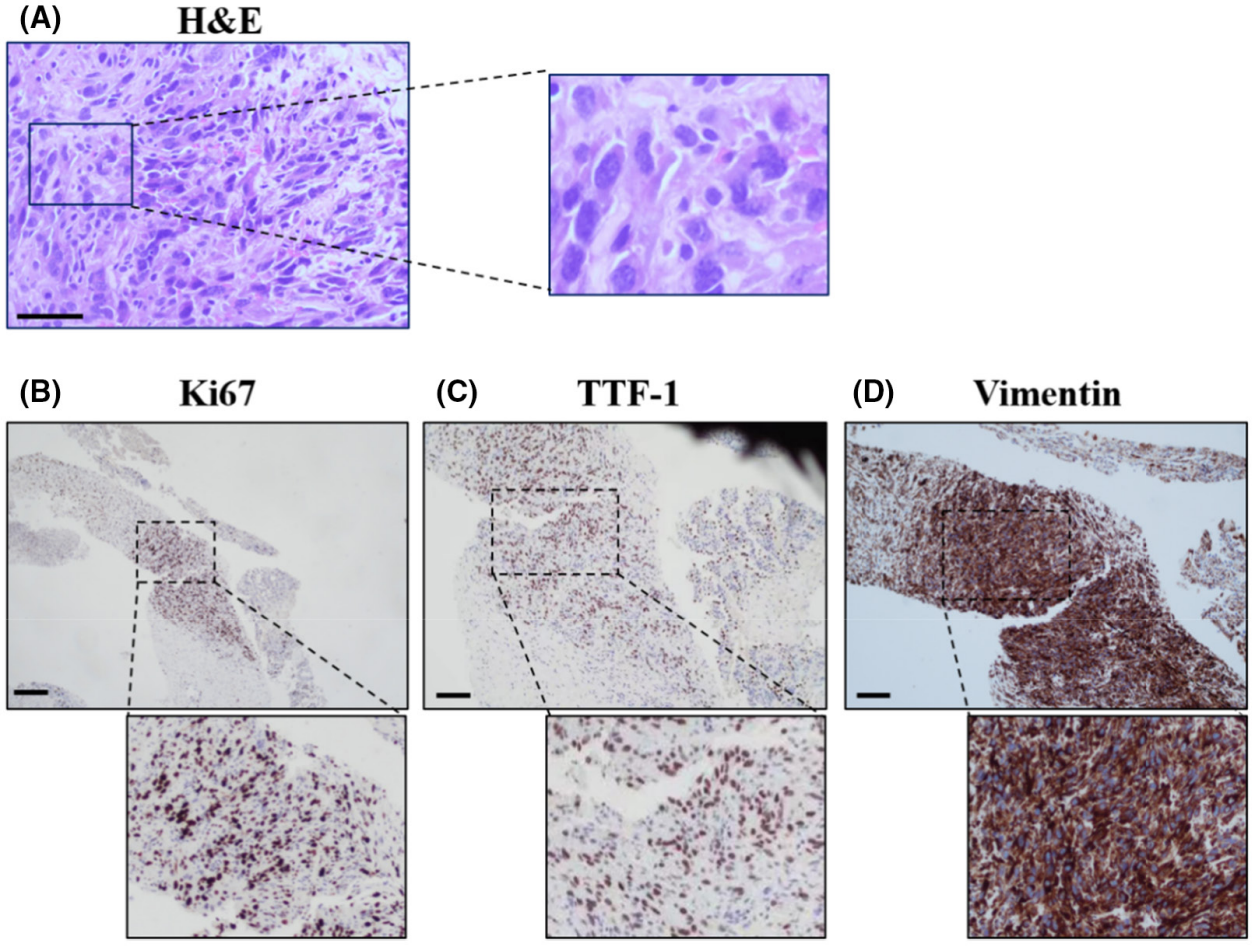
Characteristics of hematoxylin & eosin (H&E) and immunohistochemistry (IHC) staining. (A) Pathological puncture of right lung mass, H&E staining indicated that hyperplasia of the alveolar epithelial was detected in the lesion of the pulmonary tissue, histiocyte reaction emerged in the alveolar cavity, combined with focal necrosis, interstitial fibrosis, and very few atypical cells occurred in the interstitium, with deeply dyed and irregular nucleoli, heteromorphic cells were spindle‐shaped and arranged in a sarcomatous pattern. Scale bar, 100 μm. (B–D) IHC staining suggested the tumor cells stain positively for markers, including, Ki67 (65.8%+), TTF‐1 (78%+), Vimentin (93.4%+), Scale bars 50 μm.

**FIGURE 3:**
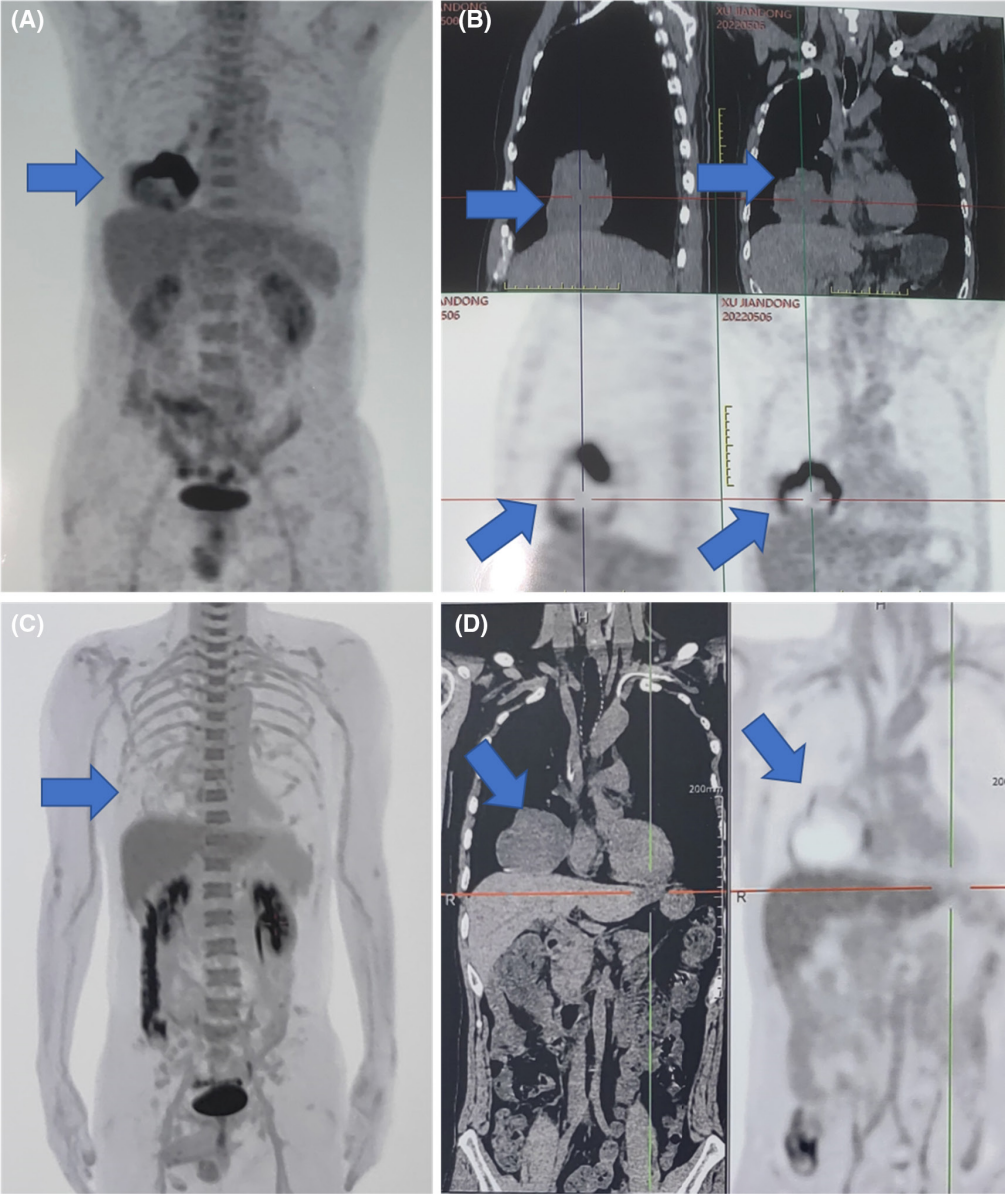
The features of PET‐CT corresponding to the patient. (A,B) A 73.2 × 52.0 mm cystic solid mass was screened in the oblique fissure of the lower lobe of the right lung. The boundary is clear. The average CT value is about 28.2 HU. The margin is accompanied by abnormal increase in glucose metabolism. The maximum SUV value is about 3.2. (C,D) A 79.8 × 65.0 mm cystic solid mass was screened in the oblique fissure of the lower lobe of the right lung. The boundary is clear. The average CT value is about 24.8HU. The margin is accompanied by abnormal increase in glucose metabolism. The maximum SUV value is about 3.2.

Unfortunately, CT revealed a clear pulmonary mass that almost filled the right interlobar fissure area (Figure 1A,B), which was also confirmed by PET‐CT (Figure 3A,B). Reviewing the medical history, a small subpleural pulmonary nodule, the diameter of which was approximately 5 mm, was detected by the chest CT in the patient's right lower lobe oblique fissure in our hospital on July 3, 2020 (Figure ^S1^A,B), but not attracted attention at that moment. After performing a pathological puncture on the right lung mass, bloody pleural effusion and tumor heteromorphic cells were discovered. Pathologically, Hematoxylin & eosin (H&E) (Figure 2A) and immunohistochemistry (IHC) staining (Figure 2B **–**D) (Figure S2A**–**G) suggested PSC. Simultaneously, extensive panel‐based genetic testing, and next‐generation sequencing (NGS), showed that ROS1 was overexpressed in this PSC patient. While, immune‐checkpoint and programmed death ligand‐1 (PD‐L1) were performed TPS (tumor proportion score) and CPS (combined positive score) were 50% and 55%, respectively. Collectively, all these results indicated and diagnosed as PSC (right lobe), subtyped as spindle cell carcinoma, non‐squamous, TNM Classification was T4NxM1a, Stage IVa. Consequently, following a multidisciplinary consultation, chemotherapy, antiangiogenesis, and immunochemotherapy were planned for the patient, including Apealea (paclitaxel micellar) (480 mg, intravenous injection, Day‐1) + Cisplatin (40 mg, intravenous injection, per day, Days 1–3 of a 21‐day cycle) + Anlotinib (12 mg, oral, per day, Days 1–14 of a 21‐day cycle) + Crizotinib (250 mg, oral, twice a day).

A re‐examination of PET‐CT revealed that after three cycles of the treatment, the lesion's glucose metabolism was significantly lower than before (Figure 3C,D). Based on this situation, the patient underwent the treatment of thoracoscopic right middle and lower lobectomy plus mediastinal lymph node dissection after discussion, which was completed as R0 resection, classification was T4N1M1a, Stage IVa. However, a severe pulmonary infection happened in the patient 2 weeks post‐operation (Figure 1C,D). A second procedure, called thoracoscopic empyema fibrous plate stripping plus thoracic irrigation and drainage, was then carried out. Shocking discovery, extensive pleural metastases were found intraoperatively, sizes ranging from 5 mm to 15 mm, and additionally, pathological biopsy was also performed which was in line with previous results but even worse (Figure S3A**–**L). No more soon, the patient developed a rapid, extensive tumor metastasis (Figure S1C,D), and in a poor general nutritional status. Although antiangiogenetic‐ and immunochemical‐therapy (Anlotinib + Crizotinib) which was stopped during this two‐operation period, was carried out again, it did not play a good job and not limited the progress of the tumor (Figure S1E–F), leading to widespread metastasis followed by multiple organ failures, eventually causing death.

In this case, this patient's condition appears to be better before operation, which may be linked to the performance of chemo‐, antiangiogenetic‐, and immunochemical therapy. However, the deterioration of this PSC was very rapid, especially after surgical treatments which seem to accelerate the death of the patient. Nevertheless, the diagnosis and treatment of this patient is still controversial.

## 3 DISCUSSION

PSC is a rare subtype of NSCLC, accounting for less than 1% of all lung cancers, with a poor prognosis.^7^, ^8^ It can metastasize through the lymph nodes and even to the bones, adrenal glands, liver, and brain. Despite the progress of imaging, the use of CT, MRI, and even PET‐CT examination methods are helpful in the diagnosis of PSC, but puncture biopsy is the best strategy to obtain pathological and genetic testing, whilst biopsy‐specific can provide the pathological basis for the immune checkpoint at the same time.^9^ This PSC patient's genetic testing (NGS) revealed ROS1 positivity, which inhibited nodule growth and improved symptoms after treatment with a ROS1 inhibitor (Crizotinib),^10^ allowing the patient to undergo surgery. However, when this patient discovered a small pulmonary nodule first 2 years ago, he did not insist on follow‐up and review, resulting in the loss of the best opportunity for surgery.^11^ Sincerely, despite receiving chemotherapy, antiangiogenetic, and immunochemical therapy, as well as having undergone two operations, the patient's condition rapidly deteriorated due to postoperative recurrence and metastasis. Our team once questioned whether the patient needed surgery after receiving chemotherapy, antiangiogenetic therapy, and immunochemotherapy as well as whether it was a mistake to stop taking Anlotinib and Crizotinib during the time leading up to surgery.

As previously reported that adjuvant chemotherapy was significantly associated with overall survival (OS) in resectable PSC patients,^2^ a 5‐year survival rate may be increased by approximately 5%.^12^ However, for advanced Stage‐PSC(IVa), non‐squamous, the effect of radiotherapy and chemotherapy is not so sensitive,^10^ making the treatment difficult. The operation was not the first line of treatment for PSC in Stage IVa.^11^, ^13^ The NCCN NSCLC Panel recommended atezolizumab in combination with bevacizumab/carboplatin (or cisplatin)/paclitaxel (ABCP) as first‐line therapy options for some patients with metastatic non‐squamous NSCLC in contrast to bevacizumab/chemotherapy based on phase III randomized trial.^11^ Apealea and Cisplatin were selected for chemotherapy. Together, since ROS1 was overexpressed in this patient, a combination of chemotherapy, targeted angiogenesis, and precise targeted‐ROS1 immunochemotherapy prevented the progress of this PSC preoperational, while the operation exacerbated deterioration. Therefore, extensive panel‐based genetic testing may deeper affect the efficacy in prognosis for PSC patients.

In NSCLC, *KRAS* mutations, especially transversion mutations, were often found in smokers, while *EGFR*, *ALK*, *ROS1*, and *RET* mutations or translocations might be more common in light smokers or non‐smokers, other alterations such as *TP53*, *NRAS*, and *MAP2K1* are also more common in smokers.^1^ Epidemiological data indicates that *ROS1* gene rearrangement occurs in approximately 1%–2% of NSCLC patients.^14^ This contradicts the fact that our patient has smoked for a long time, but the overexpression of ROS1 rather than TP53 or NRAS may be a plausible explanation for the early success of the patient's chemotherapy, antiangiogenic therapy, and targeted therapy.

Previous research suggested that TTF‐1 and Napsin A, both of which had a sensitivity of about 80% and were more easily evaluated as a nuclear stain, were well‐established markers for the identification of adenocarcinoma differentiation.^1^, ^15^, ^16^ This patient's IHC results were negative because Syn was not well expressed (Figure S2G) (Figure S3J). P40 has been reported as the most specific and sensitive marker for diagnosing squamous cell differentiation.^15^ This patient's classification of PSC was in line with 2021 WHO Classification of Lung Tumors,^1^ TTF‐1 is positive (Figure 2C) (Figure S3K), but Napsin A (Figure S2) (Figure S3G) and p40 are negative (Figure S2F) (Figure S3H), suggested that PSC was poorly differentiated NSCLC, favor adenocarcinoma.^1^ Although the Ki 67 proliferation index is not an essential criterion in the 2021 WHO classification, for PSC, it can be introduced as an ideal criterion and included in the pathological report with better diagnostic and therapeutic value, which may also be closely related to cancer metastasis.^1^, ^17^ However, all of the positive results are based on new‐generation detection methods, including NGS and biomarker of immuno‐checkpoint, etc., which are significant clinically for patients with advanced NSCLC and can be targeted more for neoadjuvant chemotherapy or immunotherapy.^18^ At one cancer center, tissue‐based NGS and liquid biopsy testing for patients with advanced NSCLC to improve the diagnostic process. Combining these two techniques has greater clinical value for patient diagnosis and the detection of complete biomarkers during disease progression.^19^

Although tumor types change over time, most of them depend on accurately distinguishing subtypes of NSCLC, such as squamous cell carcinoma and non‐squamous cell non‐small cell carcinoma. Although classical genes like epidermal growth factor receptor (EGFR) and anaplastic lymphoma kinase (ALK) have few targeted mutations in PSC, PD‐L1 overexpression was frequently observed.^20^ Therefore, reasonable and effective molecular testing, including molecular typing selection of corresponding genes and PD‐L1 detection, could be helpful for the treatment of PSC patients.^1^, ^6^ As previously stated, this patient's TPS and CPS (PD‐L1) expression levels were 50% and 55%, respectively. In NSCLC, in most cases, only PD‐L1 expression on tumor cells (fraction of tumor proportion; percentage of positive cells expressing membrane staining) was associated with a predictive biomarker of immune checkpoint inhibitor therapy.^21^ Moreover, although PD‐L1 detection is an imperfect predictor of the clinical efficacy of immune checkpoint inhibitors, currently, it is still the most important predictor of first‐line immuno‐targeted therapy.^22^

A recent study from one of China's larger medical centers found that neoadjuvant chemotherapy was significantly associated with better survival and that it should be recommended for surgically treated PSC patients, particularly those with advanced cancer, younger age, or a higher BMI.^2^ Additionally, Abdallah et al.^23^ conducted a retrospective study using the National Cancer Database. The results showed that neoadjuvant chemotherapy had long‐term survival advantages for PSC patients with Stage II and III, but did not benefit patients with Stage I. While Chaft^24^ reported that neoadjuvant chemotherapy is only effective in relatively advanced PSC (Stage IIb–IIIa vs. Ib–IIa). As a result, when combined with the current patient's condition, we have enough evidence to continue giving this patient neoadjuvant chemotherapy and immuno‐targeted therapy to improve his survival.

## 4 CONCLUSIONS

Collectively, surgery may benefit patients with resectable PSC, early detection and diagnosis are still the best way to obtain the chance of radical surgery. While chemo‐, antiangiogenetic‐ and immunochemical‐therapy may be useful in controlling the progression of advanced PSC, relieving symptoms, and extending survival. However, surgical resection after chemo‐, antiangiogenetic‐ and/or immunochemical‐therapy must be carefully considered in light of the patient's unique situation. surgical resection after adjuvant therapy must be carefully considered in light of the patient's unique situation. In particular, accurate tumor cell genetic testing or molecular pathological diagnosis plays a key role in the individualized treatment of PSC, which could benefit patients with advanced PSC.

## AUTHOR CONTRIBUTIONS

**Wei Sun:** Funding acquisition; investigation; supervision. **Xinlian Wang:** Conceptualization; validation. **Qifeng Shi:** Investigation; methodology; resources. **Xiao Li:** Conceptualization; supervision. **Chaobo Chen:** Conceptualization; formal analysis; writing – original draft; writing – review and editing.

## FUNDING INFORMATION

This study was financially supported by a research project of Wuxi Municipal Health Commission (Major, No. Z202113).

## CONFLICT OF INTEREST STATEMENT

The authors declare no competing interests.

## ETHICS STATEMENT

The study was approved by the Ethics Committee of the Wuxi Xishan People's Hospital, No. xs2022ky012. The committee decided not to require individual consent.

## CONSENT

Written informed consent was obtained from the patient to publish this report in accordance with the journal's patient consent policy.

## Supporting information


^**Figure S1**^

## Data Availability

The data that support the findings of this study are available from the corresponding author, Chaobo Chen, bobo19820106@gmail.com, on special request.
