# Supplementary material for: Comprehensive treatment for ROS1 ‐overexpressed pulmonary sarcomatoid carcinoma: A case report
Source: Clin Case Rep. 2023 May 18;11(5):e7365. doi: 10.1002/ccr3.7365 (PMC10196425; doi:10.1002/ccr3.7365)
Supplement: Supplementary file 1 — Figure S1 [file CCR3-11-e7365-s001.pdf]

SUPPLEMENTARY MATERIALS

Comprehensive treatment for ROS1-overexpressed pulmonary sarcomatoid carcinoma: a case report

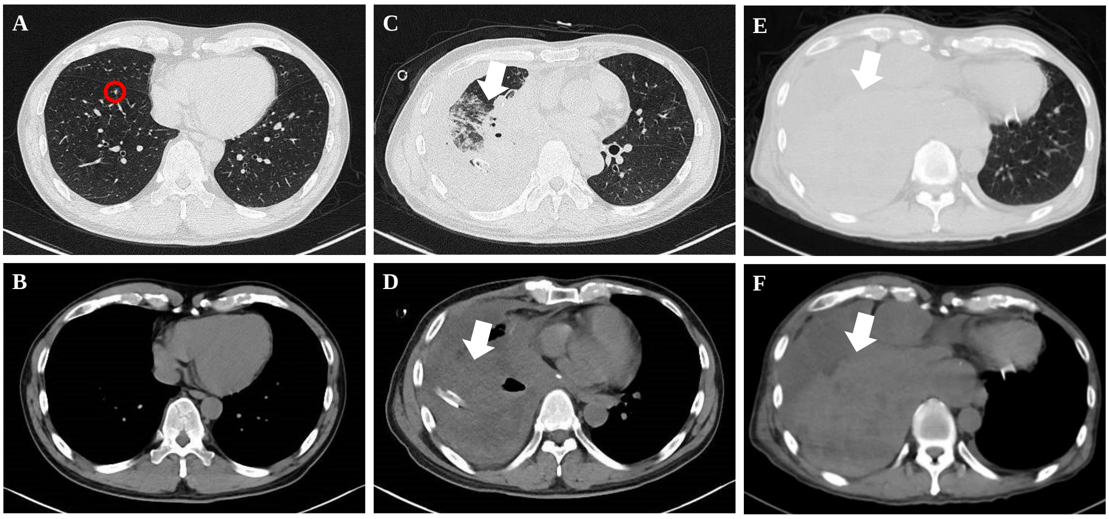

Figure S1. The features of the chest CT image correspond to the patient's condition. **(A)** A subpleural pulmonary nodule of the right inferior lobe oblique fissure as shown in the red circle, the diameter of which was 5 mm, was detected in the lung window. **(B)** In the mediastinal window, there were no visibly noticeable nodules. **(C-D)** On the chest CT image following the second procedure, the arrow indicates that the right lung infection was accompanied by partial distension and a small amount of pneumothorax. **(E-F)** Recurrent PSC was detected postoperation two months, and multiple tumors occupied nearly the whole right thoracic cavity as the arrow show.

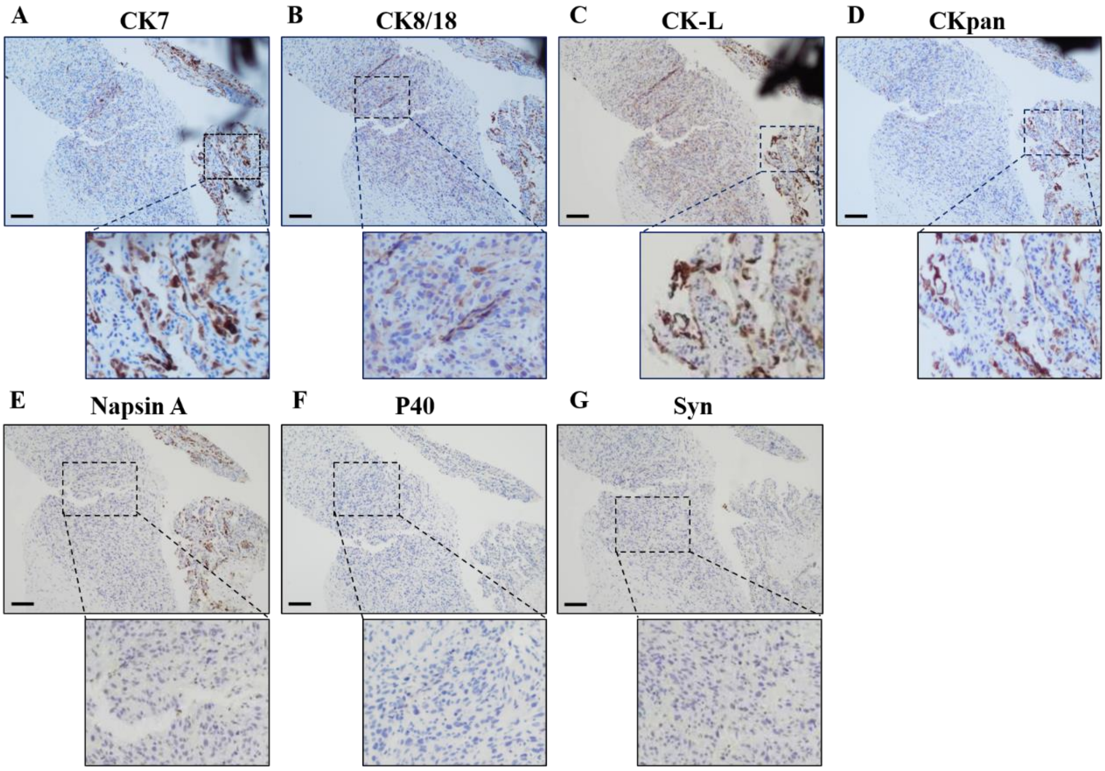

Figure S2. **Characteristics of hematoxylin & eosin (H&E) and immunohistochemistry (IHC) staining. (A-G)** IHC staining suggested the tumor cells stain positively for markers, including CK7 (16.1%+), CR8/18 (14.2%+), CK-L (15.6%+), CKpan (13%+), Napsin A (-), P40 (-), Syn (-), Scale bars 50μm.

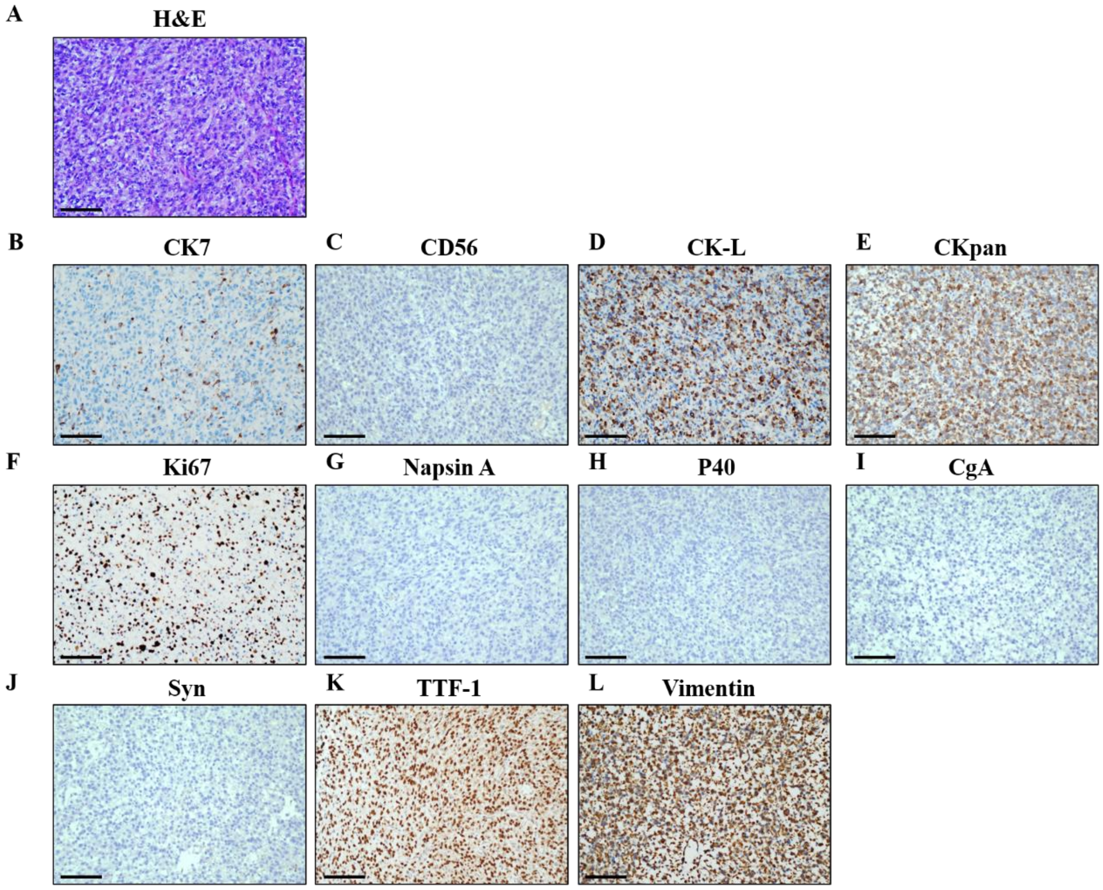

Figure S3. Characteristics of hematoxylin & eosin (H&E) and immunohistochemistry staining in the second surgery. (A), H&E staining indicated that cancer cell nests were clustered, with obvious atypia, with obviously enlarged hyperchromatic nuclei, Scale bar of 100 μm. **(B-L)** Immunohistochemistry staining suggested the cancer cells stain positively for markers, including CK7 (19.2%+), CD56 (-), CK-L (43.3%+), CKpan (34.5%+), Ki67 (80%+), Napsin A (-), P40 (-), CgA (-), Syn (-), TTF-1(86%+), Vimentin (96.7%+), Scale bars 100 μm.
